# The importance of cross-disciplinary research to combat antimicrobial resistance: introducing a new pop-up journal, *X-AMR*

**DOI:** 10.1099/mic.0.000684

**Published:** 2018-06-22

**Authors:** Gwen Knight, Helen Lambert, Edward Feil, Mark Holmes, Jodi Lindsay

**Affiliations:** ^1^​ Centre for the Mathematical Modelling of Infectious Diseases, Department of Infectious Disease Epidemiology, Faculty of Epidemiology and Population Health, LSHTM, London, UK; ^2^​ Department of Population Health Sciences, Bristol Medical School, University of Bristol, Bristol, UK; ^3^​ The Milner Centre for Evolution, Department of Biology and Biochemistry, University of Bath, Bath, UK; ^4^​ Department of Veterinary Medicine, Cambridge, UK; ^5^​ Institute of Infection and Immunity, St George's, University of London, London, UK

**Keywords:** cross-disciplinary, X-AMR, antimicrobial resistance, journal

Antimicrobial resistance (AMR) is a cross-disciplinary issue, with ground-breaking studies currently bringing together clinicians and modellers, veterinary and soil scientists, microbiologists and anthropologists. Yet finding a home for the unique publications from this research is often difficult. The Microbiology Society is providing such a home with a new pop-up journal for cross-disciplinary research on antimicrobial resistance: *X-AMR*. This initiative has been inspired by the recent prioritization of AMR as a global health issue by the World Health Organisation and UN Global Health Assembly [1, 2], collaborative efforts by research funders focusing on interventions underpinned by cross-disciplinary collaboration [3–5], as well as a desire to support an emerging community of interdisciplinary researchers. By providing a home for high-quality outputs of these essential cross-disciplinary collaborations, we aim to improve AMR research by uniting the many strands into a stronger whole.

Different disciplines have different working practices, logistical issues, language and scientific norms. Outcomes of research can be interpreted or applied in different ways. However, there is enormous value in working together to overcome these issues to fight the growing impact of AMR on human and animal health. For example, social sciences, mathematical modelling and One Health studies can offer unique and crucial insights into develop interventions to reduce AMR.

Antimicrobial resistance is a social problem [6]; its drivers and consequences are socially patterned and social science research can provide crucial insights into its social, cultural, economic and organizational determinants, without attention to which efforts to limit AMR are likely to fail. For example, research with doctors and patients reveals how perceptions of threat and uncertainty drive parent consultation and clinician prescribing of antibiotics for children with respiratory tract infections [7]. Ethnographic work in hospital settings has demonstrated the influence of organizational culture and status hierarchies on antimicrobial stewardship [8]. Qualitative work on farms and with veterinarians has identified the differing values afforded to antimicrobial therapy as forms of livestock care that require attention if interventions to limit AMR in agriculture are to succeed [9].

Mathematical modelling can provide a logical framework to underpin and combine distinct components of cross-disciplinary projects. This is especially key for AMR research where the multiple factors interact in complex ways. For example, mathematical modelling can be used to explore underlying mechanisms that drive basic microbiology as well as trends in drug resistance infection incidence [10]. This can guide new experimental design and highlight transmission hotspots for targeting interventions. Importantly, as AMR is a public health problem, modelling can be used to compare interventions for control across settings, predict likely impact and explore reasons for observed dynamics (when interventions fail or succeed). As such, mathematical models are often crucial tools for AMR research but, when combined with multifaceted data analysis, such projects commonly result in papers that have no single-theme publication home nor single audience. Our AMR pop-up journal provides this home by encouraging publishing papers of a cross-disciplinary nature.

Effective surveillance and management of AMR requires a synthetic approach that incorporates a broad range of reservoirs of resistant bacteria, viruses, parasites and fungi. The ‘One Health’ perspective acknowledges that conventional compartmentalization of clinical, veterinary, agricultural and environmental settings belies the interconnectedness of the microbial ecosystem, and that management practices should factor in potential risks extending beyond the sector in which they are applied. Prudent antibiotic stewardship in agriculture and aquaculture can help mitigate the emergence and spread of AMR genes and resistant microorganisms in clinical settings. Moreover, a greater understanding of how microbial communities in soil or water are perturbed due to anthropogenic exposure to antimicrobials will help inform improved policy guidelines and risk assessments.

There are many other scientific approaches that can also contribute to reducing AMR, including the design and discovery of new antimicrobials. The scope for *X-AMR* has deliberately been kept broad and we are open to new interpretations or conceptualizations of this emerging interdisciplinary field. We also encourage the submission of well-referenced reviews or opinion pieces addressing major unanswered questions and challenges in the field. In addition to providing a home for cross-disciplinary cutting-edge papers, this pop-up journal aims to build an AMR research community and a platform for education. The new interventions desperately needed for AMR control have to consider a range of factors from the microbiological to the anthropological. By providing a forum for high-quality research that cuts across these topic areas, we intend to support the education of a new generation of AMR researchers who have unique knowledge over this wide range of interplaying themes.

The Microbiology Society publishes four journals that have traditionally included AMR research. Authors can choose to submit their research to *Microbiology*, *Journal of General Virology*, *Microbial Genomics* or the *Journal of Medical Microbiology*. Authors can select the most suitable match for their AMR manuscript to any one of these journals; however, please contact the Microbiology Society’s editorial staff at x-amr@microbiologysociety.org if you are not sure which journal to submit to. Articles will be peer reviewed by the parent journal as usual; cross-disciplinary AMR articles that do not normally fall within the scope of our current journals will be assessed by the *X-AMR* guest editors. All accepted articles will be published in one of the four participating journals and will be available as a collection dedicated to AMR. This site will curate all articles submitted for this special pop-up journal, as well as other AMR content from all our journals. All articles will be published on PubMed and PubMed Central if authors opt for open access.

Please mention ‘Submission to *X-AMR*’ in your cover letter when submitting your paper, as this will ensure that the paper is assigned to the relevant editor. We welcome papers from any field of AMR and particularly those with a cross-disciplinary or interdisciplinary approach, and aim to provide one platform to publish all accepted AMR content submitted to us. We look forward to receiving your manuscripts.

## References

[1] WHO Global action plan on antimicrobial resistance. www.who.int/antimicrobial-resistance/global-action-plan/en/.

[2] (2016). United Nations General Assembly (UNGA) high-level meeting on antimicrobial resistance (AMR), September.

[3] Wellcome Trust Sustaining Global action on antimicrobial resistance. https://wellcome.ac.uk/sites/default/files/sustaining-global-action-on-antimicrobial-resistance.pdf.

[4] Joint Programming Initiative on Antimicrobial Resistance (JPIAMR). www.jpiamr.eu/.

[5] Tackling AMR – A Cross Council Initiative. www.mrc.ac.uk/research/initiatives/antimicrobial-resistance/tackling-amr-a-cross-council-initiative/.

[6] Smith R (2015). Antimicrobial resistance is a social problem requiring a social solution. BMJ.

[7] Cabral C, Lucas PJ, Ingram J, Hay AD, Horwood J (2015). "It's safer to …" parent consulting and clinician antibiotic prescribing decisions for children with respiratory tract infections: an analysis across four qualitative studies. Soc Sci Med.

[8] Charani E, Ahmad R, Tarrant C, Birgand G, Leather A (2017). Opportunities for system level improvement in antibiotic use across the surgical pathway. Int J Infect Dis.

[9] Swinkels JM, Hilkens A, Zoche-Golob V, Krömker V, Buddiger M (2015). Social influences on the duration of antibiotic treatment of clinical mastitis in dairy cows. J Dairy Sci.

[10] van Kleef E, Robotham JV, Jit M, Deeny SR, Edmunds WJ (2013). Modelling the transmission of healthcare associated infections: a systematic review. BMC Infect Dis.

